# Back to The Fusion: Mitofusin-2 in Alzheimer’s Disease

**DOI:** 10.3390/jcm9010126

**Published:** 2020-01-02

**Authors:** Giulia Sita, Patrizia Hrelia, Agnese Graziosi, Fabiana Morroni

**Affiliations:** Department of Pharmacy and BioTechnology, Alma Mater Studiorum—University of Bologna, via Irnerio 48, 40126 Bologna, Italy; giulia.sita2@unibo.it (G.S.); agnese.graziosi2@unibo.it (A.G.); fabiana.morroni@unibo.it (F.M.)

**Keywords:** Mitofusin 2, Alzheimer’s disease, endoplasmic reticulum, neuroprotection

## Abstract

Mitochondria are dynamic organelles that undergo constant fission and fusion. Mitochondria dysfunction underlies several human disorders, including Alzheimer’s disease (AD). Preservation of mitochondrial dynamics is fundamental for regulating the organelle’s functions. Several proteins participate in the regulation of mitochondrial morphology and networks, and among these, Mitofusin 2 (Mfn2) has been extensively studied. This review focuses on the role of Mfn2 in mitochondrial dynamics and in the crosstalk between mitochondria and the endoplasmic reticulum, in particular in AD. Understanding how this protein may be related to AD pathogenesis will provide essential information for the development of therapies for diseases linked to disturbed mitochondrial dynamics, as in AD.

## 1. Introduction

Mitochondria are the power plants of the cell. These organelles are involved in the synthesis of adenosine triphosphate (ATP), and they are responsible for the balance of nutrient storage for energy production. Moreover, mitochondria have a fundamental role in the preservation of the cell’s redox balance [1]. In the cell, these organelles form a dynamic and connected system in association with the endoplasmic reticulum (ER), which is biochemically and physically linked with the outer membrane of mitochondria (OMM). These connections are not arbitrary but occur in specific regions of the ER membrane called mitochondria-associated ER membranes (MAM) [2]. Among the numerous proteins involved in the link between ER and mitochondria, mitofusin-1 and -2 (Mfn1 and Mfn2), inositol 1,4,5-triphosphate receptor 3 (IP3R3), and voltage-dependent anion channel 1 (VDAC1) undoubtedly play key roles [3,4]. While both Mfn1 and Mfn2 occur in the OMM, only Mfn2 is located in the MAM [5]. Mfn1 and Mfn2, together with the optic atrophy-1 (OPA1) protein, are three GTPase dynamin-like proteins that mediate mitochondrial fusion in the inner and outer membranes of mitochondria [6]. The mitofusins are transmembrane proteins that form homo- and heterodimers when the fusion process is initialized, extruding the N-terminal GTPase domain that faces the cytoplasm. They use their GTPase activity to fuse the OMMs together, while the OPA1 protein controls the fusion of the inner membranes of mitochondria [7,8]. If the mitochondrial fusion ability is impaired, the organelles show fragmentation and the cell is more vulnerable to apoptosis. In this view, the fusion process may be considered as a protective mechanism [9]. On the other hand, mitochondrial fission is important for the sequestration and elimination of damaged mitochondria [10]. Several GTPase proteins participate in the fission mechanism, such as the dynamin-related protein 1 (Drp1) and the mitochondrial fission 1 protein (Fis1), that oligomerizes into a large ring-like complex surrounding the fission site along the OMM to obtain two daughter mitochondria [11].

**Figure 1:**
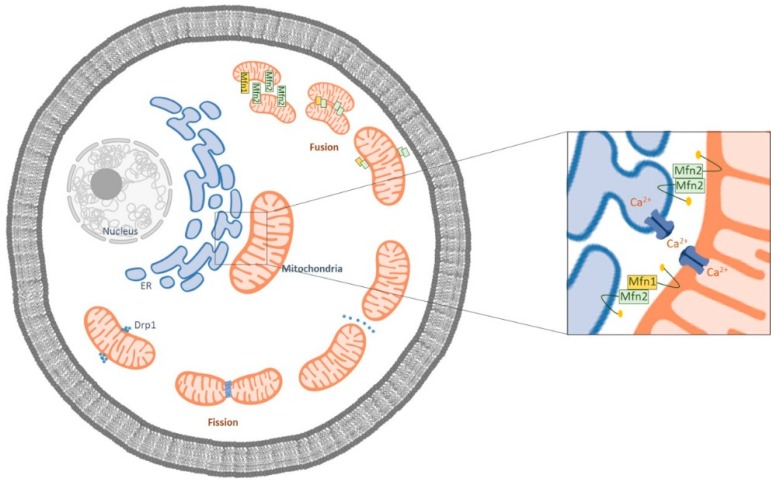
Schematic representation of the mitochondrial fusion and fission processes. Mfn1 is localized in the outer membrane of mitochondria (OMM), and Mfn2 is localized both at the OMM and at the endoplasmic reticulum ER. The ER Mfn2 binds to mitochondrial Mfn1 and Mfn2, regulating ER morphology and leading to the formation of interconnected mitochondria. In the fission process, Drp1 is mobilized to the OMM from the cytosol and is localized to the sites of organelle division.

Mitochondrial fission and fusion are maintained in a critical balance susceptible to the physiological and pathophysiological changes within the cell (Figure 1) [12].

Indeed, when mitochondrial dynamics are impaired, the organelle’s functions, including ATP production, calcium (Ca^2+^) flux, reactive oxygen species (ROS) formation, and apoptosis regulation, are compromised [13]. In particular, it seems that an increase in mitochondrial fragmentation is connected to ROS production. For example, prolonged exposure to high-glucose conditions results in increased ROS production, which could be counteracted by blocking the fission process in the mitochondria [14]. Even though the exact mechanisms by which ROS overproduction mediates mitochondrial dynamics are not fully understood; impairments in both mitochondrial ultrastructure and functions are apparently involved. Indeed, failures in mitochondrial fission and fusion can contribute to ultrastructural deficits, impairing also the mitochondrial membrane potential [15,16]. Therefore, the balance between mitochondrial fission and fusion can be deregulated by oxidative stress that, in turn, could further increase ROS generation.

Alzheimer’s disease (AD) is a progressive neurodegenerative disorder characterized by impaired cognitive function induced by the deposition of amyloid-β (Aβ) peptide and neurofibrillary tangles (NFTs) in the neocortex and hippocampal brain areas. Several studies have focused on the pathological causes of aggregation of Aβ and hyperphosphorylation of tau protein, without many encouraging results. Mitochondrial dysfunction, which can lead to free radical production, apoptosis, impaired glucose metabolism, and ultrastructural and mitochondrial DNA (mtDNA) alterations, is an early feature of AD pathogenesis and probably occurs prior to the development of Aβ plaque formation [17,18]. In a vicious cycle, impaired mitochondrial function leads to increased oxidative stress that enhances Aβ production [19]. In AD brains, ultrastructural alterations of the mitochondria (i.e., size and shape) could cause a failure in the fusion mechanism [20]. Moreover, similar mitochondrial changes occur in peripheral cells, such as fibroblasts, in patients with a sporadic form of AD [21]. In addition, oxidative stress is one of the hallmarks of AD pathogenesis, and the oxidative damage occurs in the early stages of the disease.

Considering the key role of oxidative stress in AD progression and mitochondrial dysfunction, the aim of the present review is to outline the recent knowledge about the role of the fusion protein Mfn2 in AD onset and evolution.

## 2. The Crosstalk between Mitochondria and ER in AD

Mitochondria are not only responsible for cellular energy production but also have a considerable role in the regulation of Ca^2+^ flux in the ER. It has been demonstrated that marked changes to intracellular Ca^2+^ signaling precede neuronal death and cognitive deterioration in AD [22]. The modulation of intracellular Ca^2+^ by the ER has been studied since an increase in cytosolic Ca^2+^ levels was observed in fibroblasts from asymptomatic patients at risk for AD after treatment with bradykinin, a G-protein-coupled receptor agonist [23]. Mitochondria play a double-faced role in oxidative stress modulation. Indeed, mitochondria represent a source of ROS, but at the same time they are an antioxidant system [24]. It is known that different proteins promote the physical and functional tethering between mitochondria and the ER and that altered activity of these proteins may be involved in disease onset. Therefore, it is not unexpected that alterations in ER–mitochondria connections can lead to the progression of diseases such as AD. Interestingly, presenilin 2 (PS2) and Mfn2 increase the number of contact sites [25]. PSs are fundamental components involved in the production of Aβ peptides and in Ca^2+^ homeostasis. Mutations in the gene encoding for PS2 are the most prevalent cause of the familial form of AD (FAD). In particular, it has been demonstrated that ER–mitochondria binding and the Ca^2+^ flux are increased in cells with a FAD mutation in PS2 [26,27]. Moreover, there is also an involvement of ER and mitochondria in the sporadic form of AD [28]. An interesting study conducted by Filadi et al. showed that PS2 requires the expression of Mfn2 to modulate ER–mitochondria tethering. Functional and biochemical evidence indicates that PS2, in wild type and FAD models, decreases the ER–mitochondria distance to promote Ca^2+^ flux through the interaction with Mfn2 at both sides of MAM domains. In contrast, Mfn2 can reduce the coupling even without PS2. Filadi et al. also highlighted that FAD-PS2 mutants are more enriched in MAMs than wild type PS2, forming more PS2–Mfn2 complexes, which could indicate several functional consequences [25]. In addition, different studies have shown that amyloid precursor protein (APP) mutants or exposure to soluble oligomeric Aβ causes changes in the expression of mitochondrial fission and fusion proteins, leading to an increase of mitochondrial fragmentation in neuronal cells along with neuronal deficits [29,30,31]. To address the critical role of disrupted mitochondrial fusion in neurodegeneration and other AD-related disorders, Jiang and colleagues knocked out Mfn2 in the hippocampus and cortex of mice and found that this ablation caused neuronal degeneration in vivo in a temporal order, and the pathological changes that they have highlighted are also seen during AD progression. Their findings do not necessarily suggest that the pathological events occur at a similar temporal order in the AD brain; however, they propose that altered mitochondrial dynamics could contribute to several pathological events in AD [29].

Mfn2 can be considered a mitochondria-shaping protein involved in the regulation of ER morphology [5]. In the ER, Mfn2 has two conformations, one that allows the interaction with the opposite Mfn’s binding site and the other that avoids possible interactions of Mfns with Mfn1 or Mfn2-partner proteins on the OMM to regulate Ca^2+^ homeostasis [7]. Moreover, silencing or ablation of Mfn2 interferes with ER morphology and disrupts the connections with mitochondria, diminishing the efficiency of Ca^2+^ uptake [5].

Increased ER–mitochondria coupling has been reported in different AD models. Hedskog et al. demonstrated a significant raise in the expression of MAM proteins and in the number of contacts between the ER and mitochondria in the postmortem AD brain. These results were also confirmed in an APP Swe/L mouse model, as well as after the treatment of primary hippocampal neurons with Aβ. Moreover, the increasing connections between the two organelles after exposure to Aβ caused an elevated flux of Ca^2+^ from the ER to mitochondria in neuroblastoma cells [32].

In a study conducted by Leal et al. in 2016, siRNA knockdown of Mfn2 increased ER–mitochondria tethering, leading to an elevated Ca^2+^ flux between the organelles. Interestingly, the downregulation of Mfn2 decreased the levels of Aβ by around 40%, impairing γ-secretase activity [33]. They showed that the increase of ER–mitochondria contacts impaired γ-secretase activity, leading to reduced Aβ levels. Thus, these findings may introduce new strategies to regulate γ-secretase activity by the modulation of MAM function and ER–mitochondria communication.

## 3. Mitochondrial Dysfunction in Alzheimer’s Disease

Normal mitochondrial activity is fundamental to maintain the correct polarity in neurons [34]. As a consequence, mitochondrial dysfunction promotes oxidative stress, aging, and neurodegeneration [24]. Moreover, Aβ overproduction increases mitochondrial fission and, simultaneously, decreases mitochondrial fusion activity, causing mitochondrial fragmentation and neuronal death [35]. In 2015, Park et al. conducted a study examining the correlation between the presence of Aβ oligomers (AβOs) and the changes observed in mitochondrial morphology in N2a cells. The results suggested that intracellular AβOs not only cause mitochondrial fragmentation, reducing the expression of the mitochondrial Mfns, but also lead to mitochondrial functional defects [36]. The authors also proposed that the decrease in Mfn2 expression levels can be considered more pathologically relevant than the decrease in Mfn1 expression in the mitochondrial fragmentation event, as demonstrated by the inhibition of AβO-mediated cell death after the overexpression of Mfn2 [36]. Furthermore, Gan et al. used cytoplasmic hybrid (cybrid) cells obtained from neuronal cells (SH-SY5Y) and mitochondria from AD or age-matched healthy human control subjects to evaluate the mechanisms that underlie AD-specific mitochondrial defects [37]. In this study, AD cybrids showed several features that contribute to the pathogenesis of AD, such as a decrease in respiratory chain activity and an increase of ROS production. In particular, Drp1 translocation to mitochondria was increased and Mfn2 expression was decreased in AD cybrid cells, suggesting an imbalance of mitochondrial dynamics, which may lead to the mitochondrial fragmentation observed in AD cybrids.

Growing evidence suggests the possibility that mitochondria can mediate or even initiate the pathogenesis of the sporadic form of AD. This hypothesis posits that mitochondrial impairment exists independently of Aβ deposition and probably precedes it [38,39]. A recent study used accelerated senescence OXYS rats, which simulate the main features of the sporadic form of AD, to explore the mitochondrial ultrastructure of pyramidal neurons of the CA1 region of the hippocampus and measured the levels of the key proteins involved in mitochondrial dynamics (Mfn1, Mfn2, and Drp1) [40]. The results highlighted a shift from mitochondrial fusion toward fission from 4 to 24 months of age, as confirmed by increasing Drp1 content and a decreasing Mfn2/Drp1 ratio. Indeed, this imbalance is a feature of several models of neurodegeneration.

Interestingly, Wang et al. proved that the expression of mitochondrial fusion proteins is reduced in the AD brain as compared to age-matched normal subjects, and especially in the hippocampal region, the region most involved in the pathology [41]. Moreover, in 2011 Manczak et al. evaluated the nature of AβOs and mitochondrial proteins in post-mortem AD brain tissues at different stages of the pathology. The results showed a reduction in the expression levels of mitochondrial fusion genes (Mfn1, Mfn2, and OPA1) and an increase of fission genes (Drp1), as compared to healthy controls [42]. Accordingly, in 12 month-old transgenic mice (Tg2576 line), mitochondrial fusion proteins were significantly decreased (Mfn1, Mfn2, and Opa1) in hippocampal tissues. These observations demonstrated that deposition of hippocampal Aβ induced alterations in mitochondrial dynamics in these mice [43]. On the other hand, the notion that the increased expression of Mfn2 leads to the inhibition of AβO deposition suggests the potential protective role of this fusion protein in AD. Accordingly, the gene for Mfn2 is located on the short arm of chromosome 1 (1p36), which has been widely documented as a locus associated with AD [44].

Mutations in the Mfn2 gene cause an autosomal dominant disease called Charcot–Marie–Tooth disease subtype 2A (CMT2A), which is characterized by motor and sensory peripheral neuropathy. Although CMT2A mainly affects the peripheral nervous system, recent evidence indicates that CMT2A patients might have brain pathologies [45,46]. Supporting this view, Kim et al. conducted a preliminary study on the Korean population to investigate the relation between Mfn2 and AD. This study was focused on one coding single nucleotide polymorphism (SNP) of the Mfn2 gene, and the results showed that the SNP analyzed (rs1042837) was significantly correlated with the risk of AD in the Korean population [6].

Recently, Zhang et al. investigated the involvement of Mfn2 deregulation and post-transcriptional regulation of microRNAs (miRNAs) in AD, using the senescence-accelerated mouse prone 8 (SAMP8) model and transfected HT-22 cells. MiRNAs are a class of small non-coding RNA molecules able to control post-transcriptional gene expression by the inhibition or the degradation of the target mRNAs. Zhang et al. confirmed that mRNA levels of Mfn2 were reduced in the cortical and hippocampal areas of SAMP8 mice, while the mRNA protein levels were decreased only in the hippocampus of SAMP8 mice. In particular, the Mfn2 protein seems to be located mainly in the CA3 subfield of the hippocampus, an area closely associated with learning and memory activities. Moreover, the authors not only proved that the expression of miRNA-195 was upregulated in the hippocampus of SAMP8 mice but also showed that it was an upstream regulator of Mfn2 expression during the evolution of AD [47]. Unfortunately, the study did not investigate in vivo the effects of miRNA-195 related to Mfn2 on cognitive function.

To the best of our knowledge, there is only one study that does not confirm what was discussed above. Xu et al. investigated the expression of mitochondrial proteins with AD progression in APP/PS1 mice and found differences in mitochondrial fusion and fission proteins as compared to age-matched C57BL/6 mice. The results showed a significant increase of fission and fusion protein (Drp1, Fis1, Mfn2) levels in 3 month-old APP/PS1 mice [48]. Interestingly, with disease progression, the level of Mfn2 expression increased compared to wild-type mice. These data suggest that aberrant mitochondrial dynamics may be considered an early event in AD progression [48].

Mild cognitive impairment (MCI) is characterized by a progressive decline of cognitive abilities and usually is considered a prodromal syndrome that ultimately leads to early dementia, including AD [49]. Interestingly, MCI patients show mitochondrial alterations comparable to those observed in AD patients [50]. Gan et al. used the cybrid model with mitochondria derived from MCI patients that revealed a significant rise in Mfn2 expression, but not in Drp1 as compared to non-MCI controls [51]. These data corroborate the hypothesis that the enhanced mitochondrial fusion could be considered an early step in AD, and altered levels of Mfn2 contribute consistently to this event. The results obtained by Gan et al. demonstrated that the upregulation of Mfn2 may represent a response to cellular stress, as also suggested by Sugioka et al. [52]. One possibility that merits further investigation is the idea that Mfn2 is upregulated in MCI; in AD, the protein is downregulated, indicating that the earliest stages of the disease represent the time window available for a potential therapy that targets Mfn2.

## 4. Mfn2 as A Potential Target in Alzheimer’s Disease

Research on AD therapies has resulted in only a few symptomatic drugs, which are not able to halt or slow the progression of the disease. Mitochondrial dynamics is crucial in neurons because it allows the replacement of the damaged mitochondria in non-proliferating cells. Mitochondria are undoubtedly constantly exposed to the oxidative stress caused by the production of ROS, and they accumulate mtDNA damage that leads to progressive energy production failure. In addition, the accumulation of misfolded proteins, such as the Aβ peptide, in mitochondria induces impairments in mitochondrial bioenergetics and dynamics and an increased sensitivity to apoptosis [53]. In this review, we have illustrated the fundamental role of Mfn2 in mitochondrial function in health and disease, in particular in AD. Recently, different authors have indicated how perturbations in mitochondrial dynamics, particularly in fission and fusion proteins, Drp1, Fis1, OPA1, and Mfn2, can contribute to neuropathology [54,55,56].

Presumably, neuroprotective strategies may target the stabilization of mitochondrial dynamics. Preclinical studies about the amelioration of mitochondrial dysfunction and associated synaptic failures, as well as cognitive impairments, have been reported for different polyphenols and other natural compounds and for some new synthetic drugs. This research aimed to identify possible targets and/or to investigate effects on specific aspects of mitochondrial function. Few studies have considered Mfn2 as a potential target for AD treatment. Gan et al. demonstrated that treatment with an antioxidant compound (probucol) significantly increased Mfn2 and decreased Drp1 expression in cybrid cells obtained from neurons containing mitochondria from human AD or MCI patients. These results showed that antioxidants are able to reverse the impaired fission and fusion dynamics in mitochondria [37,51].

An additional study conducted by Chen et al. evaluated the potential beneficial effect of treatment with icariin in hippocampal neurons obtained from triple-transgenic mice (3xTg-AD) [57]. Icariin is isolated from the Chinese herb epimedium and is used as an antirheumatic that has already shown consistent efficacy in AD models [58]. The authors demonstrated that treatment with icariin was able to reduce Drp1 and increase Mfn2 levels in AD neurons, likely maintaining healthy mitochondria through the modulation of their dynamics [57]. Moreover, Chimeh et al. elucidated the cytoprotective activity of a novel compound against oxidative damage in mouse hippocampal HT-22 cells. They observed that the molecule protects by decreasing ROS generation and mitochondrial membrane hyperpolarization. In that study, the expression of Mfn2 was increased while OPA1 expression was unchanged, thus confirming the key role of this protein in neuroprotective mechanisms [59].

Moreover, Zafeer et al. [60] evaluated the effect of trans-ferulic acid (FA) on the intracerebral-ventricular streptozocin (ICV-STZ) rat model. They found an increase and decrease in mitochondrial Drp1 and Mfn2 protein levels, respectively. As already described, a balance between both processes is essential for maintaining mitochondrial health in neurons. In their model, the decrease of Mfn2 induced mitochondrial dysfunction and apoptosis. Interestingly, FA administration was able to reduce apoptotic cell loss through the management of mitochondrial abnormalities associated with neurodegenerative damage.

To search for small molecules that inhibit mitochondrial division, Cassidy-Stone and colleagues used yeast screens of chemical libraries and identified a specific inhibitor called mdivi-1 [61]. Mdivi-1 selectively inhibits the GTPase activity of dynamin-1 (Dnm1) by blocking the self-assembly of Dnm1. It also prevents mitochondrial fission in mammalian cells by blocking Drp1 assembly. It has been demonstrated that cells treated with mdivi-1 are resistant to apoptosis upon apoptotic challenges, presumably by decreasing mitochondrial membrane permeabilization. Recently, Reddy et al. [62] demonstrated that mdivi-1 protects mitochondrial structure and function by regulating mitochondrial fission and fusion genes, such as Mfn2, in N2a cells treated with Aβ_1–42_ peptides.

The pathway that regulates Aβ production is well known; however, the regulation of this process is not fully understood. γ-secretase activity and consequent Aβ production are finely regulated by several proteins whose mechanisms need to be clarified. Mfn2 is one of these proteins, as shown by Leal et al. [33]. Although ablation of Mfn2 is not a reasonable treatment strategy because of the role of this protein in mitochondrial stability, their data showed that the modulation of ER–mitochondria interaction affects γ-secretase function. This finding may introduce new approaches to regulate γ-secretase activity besides γ-secretase inhibitors and modulators.

## 5. Conclusions

Mitochondrial function is considerably impaired in AD, and there is rising interest in understanding how to inhibit neurodegeneration by targeting altered mitochondrial activities. The accurate regulation of mitochondrial turnover, responsible for the elimination of dysfunctional mitochondria and the maintenance of functional ones in response to stresses, may be crucial to control neurodegeneration in AD. Indeed, it is likely that mitochondrial homeostasis reflects an efficient bioenergetic metabolism as well as mitochondrial dynamics—two faces of the same coin. In this context, the role of Mfn2 is essential for preserving proper mitochondrial network dynamics. Thus, novel therapeutics able to target mitochondria should have the potential to act simultaneously on the two aspects of mitochondrial function. Further studies are needed to discover new promising agents capable of selectively targeting the mitochondria, and in particular Mfn2.
